# Traditional Formula, Modern Application: Chinese Medicine Formula Sini Tang Improves Early Ventricular Remodeling and Cardiac Function after Myocardial Infarction in Rats

**DOI:** 10.1155/2014/141938

**Published:** 2014-05-25

**Authors:** Jiangang Liu, Karoline Peter, Dazhuo Shi, Lei Zhang, Guoju Dong, Dawu Zhang, Heimo Breiteneder, Johannes Jakowitsch, Yan Ma

**Affiliations:** ^1^Center of Cardiology, Xiyuan Hospital, China Academy of Chinese Medical Sciences, No. 1 Xiyuan Caochang, Haidian District, Beijing 100091, China; ^2^Molecular Research in Traditional Chinese Medicine Group, Department of Pathophysiology and Allergy Research, Vienna General Hospital, Medical University of Vienna, Waehringer Guertel 18-20, 1090 Vienna, Austria; ^3^Clinical Division of Cardiology, Department of Internal Medicine II, Vienna General Hospital, Medical University of Vienna, Waehringer Guertel 18-20, 1090 Vienna, Austria

## Abstract

Sini Tang (SNT) is a traditional Chinese herbal formula consisting of four different herbs: the root of *Aconitum carmichaelii*, the bark of *Cinnamomum cassia*, the rhizome of *Zingiber officinale*, and the root of *Glycyrrhiza uralensis*. This study aims to evaluate the improvement of early ventricular remodeling and cardiac function in myocardial infarction (MI) rats by SNT. A MI model was established by ligation of the left anterior descending coronary artery. Following treatment for 4 weeks, ultrasonic echocardiography was performed. Myocardial histopathological changes were observed using haematoxylin and eosin staining. Collagens (type I and type III), transforming growth factor-**β**1 (TGF-**β**1), and Toll-like receptors (TLR-2 and TLR-4) were measured in plasma, serum, and myocardial tissue. SNT treatment decreased the infarct size, the left ventricular cavity area/heart cavity area ratio, and the left ventricle dimension at end systole and increased the left ventricular ejection fraction. SNT reduced the levels of TLR-2 and TLR-4 in myocardial tissue significantly and decreased the collagens content in serum and in myocardial tissue. SNT could partially reduce the level of TGF-**β**1 in serum and in myocardial tissue. Our data suggest that the Chinese medicine formula SNT has the potential to improve early ventricular remodeling and cardiac function after MI.

## 1. Introduction

Myocardial infarction (MI) can be a killing disease. It is one of the major causes of heart failure (HF). Every sixth man and every seventh woman in Europe died from MI [1]. Recovery from MI is characterized by stages of initial inflammation, angiogenesis, fibroblast proliferation, and collagen deposition, followed by scar formation in the maturation [2] and remodeling phase leading to infarct area expansion and dilatation of the heart by left ventricle (LV) remodeling, and ultimately develops into chronic HF [3–5]. The current medical treatment of MI includes antithrombotic therapy, beta-blockers, lipid-lowering therapy, nitrates, calcium antagonists, angiotensin-converting enzyme inhibitors, and angiotensin receptor blockers [1]. Still, there is an urgent need for novel additional therapeutic compounds supporting either conservative pharmacological treatment or replacing it by newly identified pharmaceuticals.

Traditional Chinese medicine (TCM) has been used in China for centuries for treatment of cardiac disease and is now attracting interest in Western countries as a source of alternative or complementary therapies due to its reputed effectiveness, low cost, and relative absence of side effects. Previous studies provided scientific evidence to support the use of Chinese herbal medicine (CHM) for treating MI and HF [6–11].

We used a popular traditional Chinese herbal formula, “Sini Tang” (SNT), first described by Zhang (150–219 A.D.) in his book “Treatise on cold-induced diseases,” a medical collection from ancient China for this study [12]. In terms of TCM, SNT was described as a remedy acting on the heart and has the essential effect of recuperating the patients from collapse. It was used to treat the syndrome of displaying cold extremities, cold sweating, vomiting, and lethargy, which corresponds with clinical symptoms of MI and HF in Western medicine [13–16]. SNT used for this study consists of four Chinese medicinal herbs: the processed root of* Aconitum carmichaelii* Debeaux (aconite), the bark of* Cinnamomum cassia* (L.) J. Presl. (cinnamon), the rhizome of* Zingiber officinale* Roscoe (ginger), and the processed root of* Glycyrrhiza uralensis* Fisch. ex DC. (licorice) [17]. The SNT decoction has been used to improve blood circulation, remove blood stasis, and treat myocardial damage in Chinese clinics [2, 8, 13]. However, there is still a lack of further clinical evidence and definitive mechanisms of action to demonstrate the role of SNT in cardiovascular diseases. The purpose of this study was to evaluate the improvement of early ventricular remodeling after MI by SNT using a well-established experimental rat model of MI [18–20]. We analyzed cardiac tissue structure and function and associated pathophysiological indicators together with a number of potential biomarkers, that is, collagens (type I and type III), transforming growth factor-*β*1 (TGF-*β*), and Toll-like receptors (TLR-2 and TLR-4), which could play an important role in cardiac remodeling and wound repair after MI [21–25].

## 2. Materials and Methods

### 2.1. Ethics Statement

All animal experiments were approved by the Administrative Committee of Experimental Animal Care and Use of Xiyuan Hospital, China Academy of Chinese Medical Sciences (CACMS, permit number: CACMS/20100322), and conformed to the National Institute of Health Guide for the Care and Use of Laboratory Animals [26] and the Animal Research: Reporting* In Vivo* Experiments (ARRIVE) guidelines [27].

### 2.2. Drugs and Reagents

Fosinopril sodium (FS) (10 mg/tablet) was supplied by Sino-American Shanghai Squibb Pharmaceutical Co., Ltd., TGF-*β*1, collagen type I (col-I) and collagen type III (col-III), and TLR-2 and TLR-4 kits were provided by R&D systems, USA. Coomassie blue protein assay kit was offered by Nanjing Science and Technology Co., Ltd. The Two-Step Immunohistochemical Detection Kit was produced by Beijing Zhongshan Golden Bridge Biotechnology Co., Ltd.

### 2.3. SNT Preparation

SNT (0.5 g/g extract/crude drug, batch number 20100820) containing* Glycyrrhiza uralensis*,* Aconitum carmichaelii*,* Zingiber officinale,* and* Cinnamomum cassia* (8 : 6 : 3 : 1) was prepared at the Pharmacy Department of Xiyuan Hospital, China Academy of Traditional Chinese Medicine (Beijing, China). According to WHO General Guidelines for Methodologies on Research and Evaluation of Traditional Medicine, a sufficient number of dose levels should be used in rodents to determine the approximate lethal dose [28]. Therefore, we designed our study using two doses of SNT for MI rats, a low dose of 4.5 g/kg and a high dose of 13.5 g/kg. Herbs were soaked in drinking water (500 mL) for one hour in a clay pot at room temperature and then cooked to boiling. The decoction was performed twice by cooking gently for 30 min. Two extracts were combined, filtered, and stored at room temperature before administration.

### 2.4. Animals

Ninety (50% male and 50% female) Sprague-Dawley (SD) rats weighing 190 ± 10 g were provided by the Vital Laboratory Animal Technology Company, Beijing, China. Animals were acclimatized with a 12/12 hours light/dark cycle at a controlled room temperature of 23–25°C and a humidity of 50–70% and allowed free access to foods and water for seven days before use.

### 2.5. Animal Model of MI

Acute myocardial infarction (AMI) was induced in rats by left anterior descending artery (LAD) ligation. The surgical procedures were performed using the well-established technique [19, 29]. The rats were anaesthetized by intraperitoneal (i.p.) injection of a 1% solution of sodium pentobarbital (50 mg/kg) and placed in a supine position on a table. The preoperative recording was performed by a two-lead electrocardiogram (ECG, General Meditech Inc., Shenzhen, China). After disinfection of local skin, the chest was opened to expose the heart. The LV, aorta, and left atrium were made visible for suture placement. A 3-0 suture was placed in the anterior myocardium to occlude the left anterior descending artery (LAD). The heart was returned to its original position. The rats in the control group were sham operated with thoracotomy and cardiac exposure but without coronary artery ligation. The sternum and skin incision was closed with 7-0 sutures. Additional two-lead ECG recordings were made postoperatively. Successful ligation was confirmed by ST segment elevation in postoperative ECG, compared with preoperative ones. To prevent infection rats were given penicillin (40.000 units) by i.p. injection after operation for 3 days. Sixty surviving infarct rats were randomly divided into five groups (Table 1). The oral administration of the drugs began two days after the AMI-induction. SNT (4.5 and 13.5 g/kg) and FS (0.9 mg/kg), an angiotensin converting enzyme (ACE) inhibitor used as a positive control [30, 31], were diluted with distilled drinking water and administered orally in a volume of 10 mL/kg body weight once every morning for 4 weeks.

### 2.6. Echocardiography Assessment

Transthoracic echocardiographic studies were performed 30 days after surgery according to the method of Yin et al. [19]. Ten rats from each group were anesthetized by i.p. injection of ethyl carbamate (6 mL/kg). Rats extremities were fixed to four electrocardiography leads on the table. After cleaning the rat chest the cardiac short axis (papillary level), left ventricle end-diastolic dimension (LVDd), and left ventricle end-systolic dimension (LVDs) were measured using an ATL HDI-5000 Diagnostic Ultrasound System (Philips Ultrasound Inc., China). The values were calculated on an average of three cycles. The ejection fraction (EF) was calculated from the left ventricle end-diastolic volume (LVEDV) and the left ventricle end-systolic volume (LVESV) as EF% = [(LVEDV − LVESV)/LVEDV] × 100. The echocardiographic analysis and the data calculation were performed using a single-blind method [32, 33].

### 2.7. Histopathology: Blood Sampling and Tissue Collection

After echocardiographic measurements, the anesthetized rats were sacrificed. Blood samples were taken with a heparinized syringe from the LV cavity and centrifuged at 3000 rpm for 15 min. Plasma and sera were conserved at −80°C for further analysis. The hearts were removed from the chest, excised, and weighed for determination of the heart weight/body weight (HW/BW) ratio. Six hearts randomly assigned of each group were stored in 10% neutral formalin solution for hematoxylin eosin (H&E) staining and immunohistochemistry measurements. One heart of each group was stored at −80°C for ELISA.

### 2.8. Measurement of Myocardial Infarct Size (IS)

The pathological slice was placed under natural light and photographed in microdistance using a Canon IXUS 90IS digital camera. The microscopic color image processing system (DpxView Pro, Korea) was used to calculate the left ventricular IS (%, myocardial infarction area/left ventricular area × 100) by an investigator who was blinded to the identity of the pathological slice as described by Takagawa et al. [34].

### 2.9. Detection of Collagens and TGF-*β*1

Five of the frozen heart tissue samples of each group were randomly picked. The heart sections were weighed and 300 mg of each sample were cut into small pieces and suspended in 1 mL of saline solution. After homogenization on ice, samples were centrifuged and the supernatants were stored at −80°C for analysis. Col-I and col-III and TGF-*β*1 were measured by ELISA in serum and heart tissue samples according to the manufacturer's instructions [35, 36]. The total protein content of each tissue sample was measured with the BCA (bicinchoninic acid) Protein Assay Kit.

### 2.10. Immunohistochemistry of TLRs in Myocardial Tissue

Immunohistochemical staining for TLRs in myocardial tissue was performed using Power Vision Two-Step Histostaining Reagent (Golden Bridge International Inc., Beijing, China) as described by the manufacturer. Myocardial sections (5 *μ*m thick) were fixed in 10% neutral buffered formalin for 18 hours and then deparaffinized and rehydrated. The tissue sections were incubated in 3% H_2_O_2_ for 10 min and in endogenous peroxidase for 10 min, respectively. After washing twice in PBS buffer, the tissue sections were incubated in 10% normal goat serum with anti-TLR-2 (1 : 200) and anti-TLR-4 (1 : 800) antibodies at 37°C for 1 hour, followed by incubation with a biotinylated secondary goat anti-mouse IgG for 30 min at room temperature. The detection was performed using the DAB Liquid System (Golden Bridge International Inc., Beijing, China). Expression of Toll-like receptors TLR-2 and TLR-4 in the myocardial tissue was determined as integrated optical density (IOD) values using an image analysis system (Imagepro-Plus 6.0, Media Cybernetics, USA).

### 2.11. Statistical Analysis

All results were tested on normal distribution by aid of One-Sample Kolmogorov-Smirnov Test. Data were tabulated and presented as the mean ± standard deviation, and the significance of changes was assessed with one-way repeated measures analysis of variance (ANOVA). Bonferroni Holm test was followed for multiple comparisons. One-way ANOVA Tukey HSD test was used for pairwise multiple comparisons. A value of *P* < 0.05 was considered statistically significant. Data were analyzed using the Statistical Package for the Social Sciences (version 17, SPSS Software, SPSS Inc., Chicago, USA).

## 3. Results

### 3.1. SNT Treatment Reduced Infarct Size and Left Ventricular Cavity Area/Heart Cavity Area

**Figure 1:**
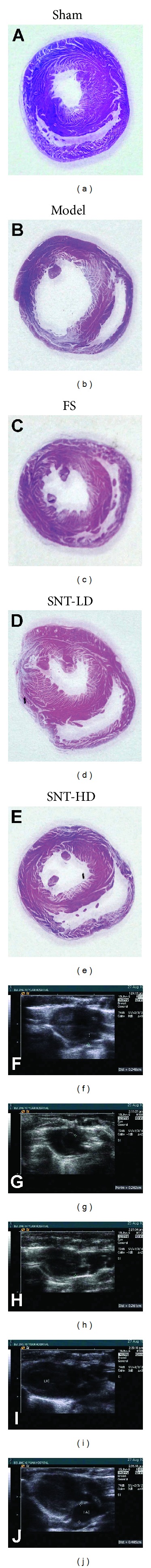
Histological and echocardiographic images. (a–e) Haematoxylin and eosin staining of midventricular cross-sections. (f–j) Two-dimensional-guided M-mode echocardiographic images of the left ventricle.

The infarct size (IS) obtained using the midline length measurement is shown in Table 2. The IS values from the FS, SNT-LD, and SNT-HD groups (23.91 ± 7.99 mm^2^, *P* < 0.01, 31.25 ± 10.68 mm^2^ and 27.81 ± 10.33 mm^2^, *P* < 0.05) were significantly smaller than from the model group (38.04 ± 8.35 mm^2^). As shown in Figures 1(a)–1(e) and Table 2, pathological H&E staining showed that the left ventricular cavity area of the model group after AMI was significantly increased by approximately 22.3% compared to the sham group (*P* < 0.01). The ventricular cavity area/heart cavity area (LVAC/HCA) ratios of the FS, SNT-HD, and SNT-LD groups (15.2%, *P* < 0.01; 18.5% and 17.3%, *P* < 0.05, resp.) were decreased significantly compared to the model group. The heart weight/body weight (HW/BW) ratio in the model group was significantly increased compared with the sham group (0.37 ± 0.07 mg/g versus 0.31 ± 0.01 mg/g, *P* < 0.01). The HW/BW ratios in the FS group (0.36 ± 0.08 mg/g) and the SNT groups (0.35 ± 0.08 mg/g and 0.36 ± 0.07 mg/g) were decreased compared with the model group, but not significantly.

### 3.2. SNT Treatment Decreased LVDd and LVDs and Improved the Cardiac Function by Increasing the EF

**Figure 2:**
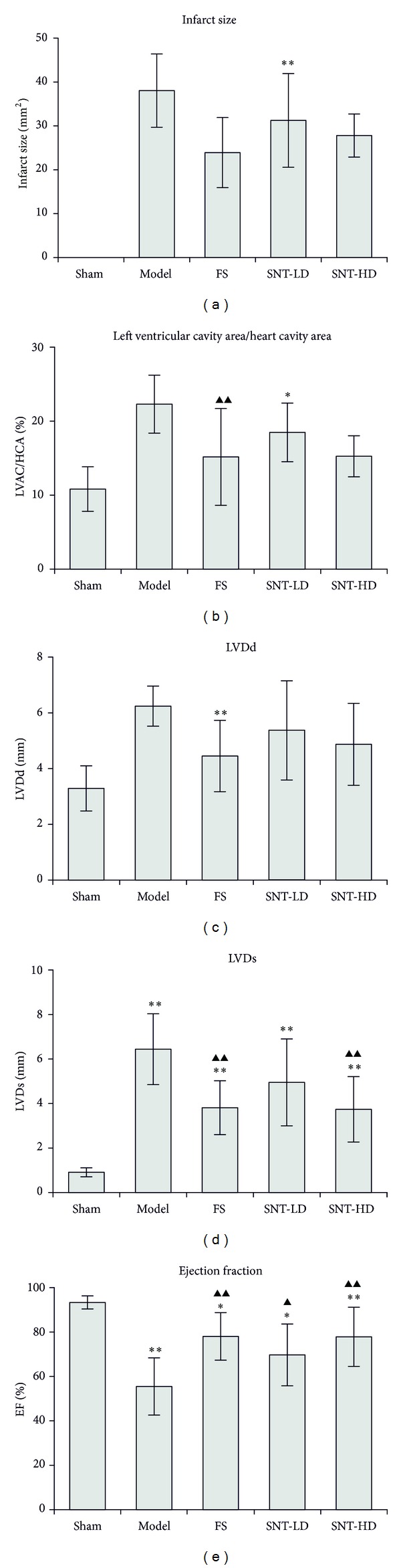
Echocardiographic parameters. (a) Infarct size results, (b) echocardiographic measurements of left ventricular dimension at end diastole (LVDd), (c) left ventricular dimension at end systole (LVDs), (d) left ventricular ejection fraction (EF), and (e) left ventricular cavity area/heart cavity area ratios (LVAC/HCA).

Four weeks after MI, ultrasound echocardiography showed a significant increase of the left ventricular dimension at end diastole (LVDd) and the left ventricular dimension at end systole (LVDs) in the model group (6.24 ± 0.72 mm versus 3.29 ± 0.81 and 6.44 ± 1.59 mm versus 0.91 ± 0.20, *P* < 0.05) compared to the sham group. The FS and SNT treatment groups exhibited significantly decreased LVDs versus the sham group (FS: 3.81 ± 1.21 mm, SNT-LD: 4.95 ± 1.95 mm, and SNT-HD: 3.74 ± 1.47 mm, *P* < 0.01). The FS treatment group exhibited significantly decreased LVDd (4.45 ± 1.28 mm versus 3.29 ± 0.81 mm, *P* < 0.01). The SNT treatment groups also showed decreased LVDd versus the sham group (SNT-LD: 5.37 ± 1.78 mm and SNT-HD: 4.87 ± 1.47 mm, *P* > 0.05), but not significantly. The left ventricular ejection fraction (EF) was significantly lower in the model group compared to the sham group (55.48 ± 12.89% versus 93.32 ± 2.94%, *P* < 0.01). The FS and SNT treatment groups showed an improved left ventricular function of the EF compared to the sham (FS: 78.03 ± 10.70%, SNT-LD: 69.69 ± 13.9%, *P* < 0.05, and SNT-HD: 77.83 ± 12.32%, *P* < 0.01) and model groups (FS and SNT-HD: *P* < 0.01, SNT-LD: *P* < 0.05). SNT treatment improved the cardiac function by increasing the EF by 23.63% (difference between sham group: 93.32% and SNT-LD: 69.69%), (Table 2 and Figures 1(f)–1(j) and 2(c)–2(e)).

### 3.3. SNT Treatment Decreased the Expression Levels of TLR-2 and TLR-4 in Myocardial Tissues

**Figure 3:**
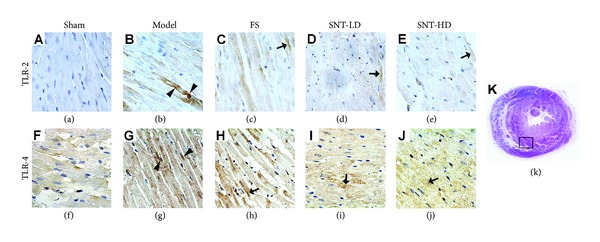
Immunohistochemical analysis of Toll-like receptors in myocardial tissue. TLR-2 (a–e) and TLR-4 (f–j) expression in early VR rats was observed in each experiment group after AMI using a 400x optical microscopy. Expression of TLRs is much stronger in the tissue of the model group in comparison to the sham group as shown by darker and lager granules (arrowheads in (b) and (g)). The FS and both SNT treatment groups showed reduced expression of TLR-2 and TLR-4 compared to the model group (arrows in (c), (d), (e), (h), (i), and (j)).

Four weeks after MI immunohistochemical staining for TLRs in myocardial tissue was performed using the Power Vision Two-Step Histostaining method. In tissues of the sham group, only a small amount of brown granules was visible indicating basal expression of TLR-2 and TLR-4 proteins. In contrast, expression of TLRs is much stronger as shown by darker and larger granules in the tissue of the model group (indicated with arrowheads in Figures 3(b) and 3(g)). The FS and both SNT treatment groups showed reduced expression of TLR-2 and TLR-4 compared to the model group (arrows in Figures 3(c) to 3(e) and 3(h) to 3(j)).

**Figure 4:**
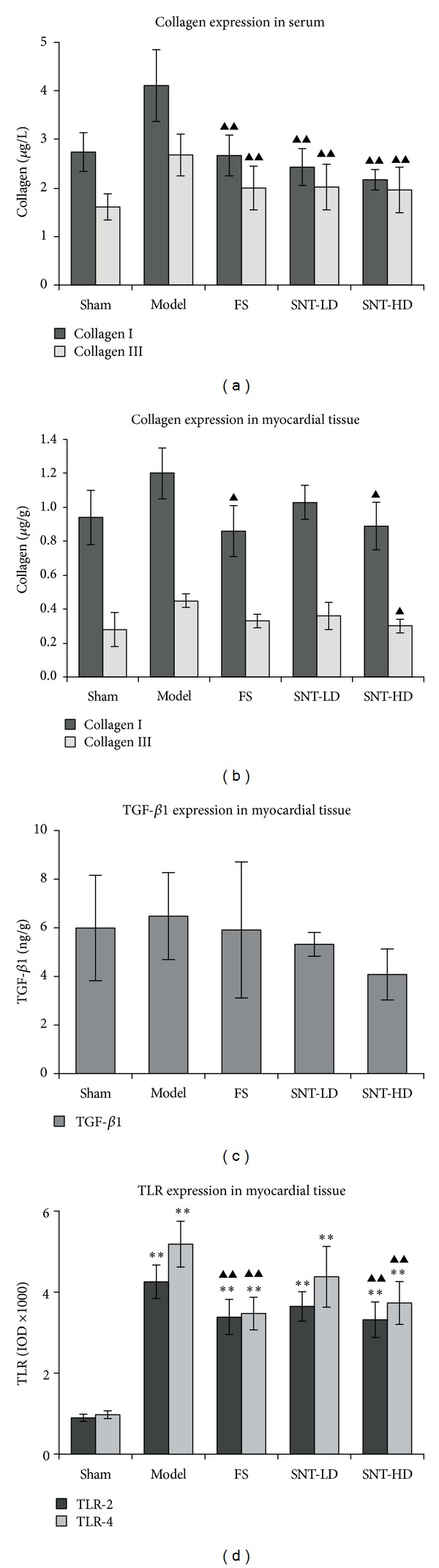
Expression levels of type I and type III collagens. Collagen I and collagen III levels in serum (a) and myocardial tissue (b), TGF-*β*1 expression levels in myocardial tissue (c), and TLR expression levels in myocardial tissue (d).

TLR-2 and TLR-4 expressions in the myocardial tissue were determined using an Imagepro-Plus Media Cybernetics system shown in Table 3 and Figure 4(d) as integrated optical density (IOD) values. In comparison to the sham group, TLR-2 and TLR-4 expressions were increased significantly in the model group (*P* < 0.01) as well as in the FS and SNT treatment groups (model: 4254.60 ± 413.66, FS: 3381.00 ± 432.17, SNT-LD: 3646.60 ± 362.37, and SNT-HD: 3316.60 ± 439.06 versus 896.40 ± 89.18 for TLR-2 expression and model: 5184.40 ± 566.30, FS: 3470.60 ± 403.33, SNT-LD: 4378.60 ± 748.49, and SNT-HD: 3732.20 ± 528.23 versus 973.00 ± 95.13 for TLR-4 expression). In comparison to the model group, TLR-2 and TLR-4 expression were decreased in all other experimental groups and significantly lower in the FS and SNT-HD treatment groups (*P* < 0.01). The SNT-HD group showed higher expression levels for both TLR-2 and TLR-4 than the SNT-LD group in our experiments.

### 3.4. SNT Treatment Reduced the Levels of Collagens and TGF-*β*1

Collagen type I and type III and TGF-*β*1 were measured by ELISA using kits from R&D Systems, USA. In serum, the levels of col-I and col-III were increased significantly in the model group compared to the sham group (col-I: 4.11 ± 0.74 *μ*g/L versus 2.74 ± 0.40 *μ*g/L, col-III: 2.68 ± 0.43 *μ*g/L versus 1.61 ± 0.27 *μ*g/L, *P* < 0.01) 4 weeks after MI. The levels of col-I and col-III in the FS and SNT treatment groups were decreased significantly compared to the model group (col-I, FS: 2.67 ± 0.42 *μ*g/L, SNT-LD: 2.43 ± 0.38 *μ*g/L, and SNT-HD: 2.17 ± 0.21 *μ*g/L versus 4.11 ± 0.74 *μ*g/L, *P* < 0.01; col-III, FS: 2.00 ± 0.45 *μ*g/L, SNT-LD: 2.02 ± 0.47 *μ*g/L, and SNT-HD: 1.96 ± 0.47 *μ*g/L versus 2.68 ± 0.43 *μ*g/L, *P* < 0.01). The levels of TGF-*β*1 in serum did not show a significant difference between the model and SNT-LD groups. The levels of TGF-*β*1 were decreased in the FS group when compared to the sham group, but not significantly (Table 4 and Figures 4(a)–4(c)).

In myocardial tissue, the levels of collagen type I and type III in the model group were decreased compared to the sham group (col-I: 1.20 ± 0.15 *μ*g/g versus 0.94 ± 0.16 *μ*g/g, col-III: 0.45 ± 0.04 *μ*g/g versus 0.28 ± 0.10 *μ*g/g, *P* < 0.05). The levels of collagen type I and type III in the FS and SNT treatment groups were decreased compared to the model group (col-I, FS: 0.86 ± 0.15 *μ*g/g, *P* < 0.01; SNT-LD: 1.03 ± 0.10 *μ*g/g; SNT-HD: 0.89 ± 0.14 *μ*g/g, *P* < 0.01, versus 1.20 ± 0.15 *μ*g/g). The level of TGF-*β*1 in serum was increased in the model group compared with the sham group (6.48 ± 1.79 ng/g versus 5.99 ± 2.17 ng/g). The levels of TGF-*β*1 were decreased in the FS and SNT groups when compared with sham and model groups, but not significantly (Table 4 and Figures 4(a)–4(c)). SNT reduced collagen matrix accumulation in the serum and the myocardial tissue following MI, which is associated with a significant improvement in systolic function (Table 2).

## 4. Discussion

Traditional Chinese medicine (TCM) is becoming an increasingly popular form of alternative or complementary medicine in Europe not only due to its reputed effectiveness, low cost, and relative absence of side effects but also due to the personalized therapy urgently needed in many countries [37, 38]. In recent years, an increasing number of studies provide scientific evidence to support the use of TCM for preventing and treating cardiovascular diseases [6–11] including SNT [15, 16]. Nevertheless, the role of TCM in cardiovascular diseases still requires further experimental evidence [39].

The Chinese herbal medicine SNT is a “cold-extremities” decoction, the representative formula of preventing and treating “cold syncope due to deficiency of the heart function” according to TCM theory. The present study indicated that SNT could play a role in treating MI caused by deficiency of the heart function.

Left ventricular remodeling is the process by which ventricular size, shape, and function are regulated by different mechanical, neurohormonal, and genetic factors. Remodeling may be physiological and adaptive during normal growth or pathological due to MI, cardiomyopathy, hypertension, or valvular heart disease [40]. After MI, the ventricle undergoes a progressive physiological and anatomical transformation. Progressive left ventricular dilation and eccentric hypertrophy, infarct scar thinning, and, ultimately, an alteration of the left ventricular geometry from a prolate ellipse to a spherical globe characterize this transformation. The structural changes in the left ventricle (infarct zone and noninfarct zone) are governed by cellular and molecular mechanisms in a pathological metamorphosis [22]. More recent concepts also include the genomic expression resulting in molecular, cellular, and interstitial changes of morphology and structure [5, 41]. In this study, we observed the morphology of the myocardial tissue with visible MI in the model and the control groups with an expansion in the total LV cavity area, an increase in thickness of the interventricular septum and free wall, and a decrease in thickness of the infarct area. The SNT treatment groups showed reduced changes in the pathological structure. Pathological H&E staining showed that the left ventricular cavity area of the model group after AMI was significantly increased by approximately 22.3% compared to the sham group (*P* < 0.01). The ventricular cavity area/heart cavity area ratios of the FS, SNT-HD, and SNT-LD treatment groups were decreased significantly by 15.2% (*P* < 0.01), 18.5% (*P* < 0.01), and 17.3% (*P* < 0.01), respectively, compared to the model group. The IS values from the FS, SNT-LD, and SNT-HD groups (23.91 ± 7.99 mm^2^, 31.25 ± 10.68 mm^2^, and 27.81 ± 10.33 mm^2^) were significantly smaller than from the model group (38.04 ± 8.35 mm^2^). SNT could decrease LVDs significantly (SNT-LD: 4.95 ± 1.95 mm versus the sham group, *P* < 0.05, and SNT-HD: 3.74 ± 1.47 mm versus the sham group, *P* < 0.01). SNT could also decrease LVDd (SNT-LD: 5.37 ± 1.78 mm and SNT-HD: 4.87 ± 1.47 mm versus the sham group, *P* > 0.05), but not significantly.

Echocardiography is the key diagnostic tool and was performed to assess LV function and volumes, valvular function, and extent of myocardial damage and to detect mechanical complications [42]. The results of our echocardiographic evaluation indicated that the rats in the SNT treated groups had a thinner wall in the infarct zone, an increased ventricular cavity area, extended circumference, wall motion abnormality, and decreased left ventricular function. The left ventricular ejection fraction (LVEF) was significantly decreased in all groups compared with the sham group (*P* < 0.05). The SNT groups showed an improved left ventricular function of the EF% (24%, *P* < 0.05) compared with the model group.

Surrounding the myocytes, the extracellular matrix (ECM) is a dynamic complex composed of structural components: collagen, especially type I and type III, and fibroblasts. Collagen type I, a fibrillar collagen which provides tensile strength, and collagen type III, an elastic collagen, are most abundant in the cardiac ECM [22]. Collagen is important in maintaining structural integrity after AMI [43]. Studies indicated that infarct expansion is associated with damage to the myocardial connective tissue matrix, including the apparent loss of collagen struts. Collagen synthesis is controlled by TGF-*β*, a member of the transforming growth factor beta superfamily of cytokines, which plays an important role in fibrosis-related cardiovascular disorders, postangioplasty restenosis, and postinfarct ventricular remodeling, all of which can lead to heart failure [44]. TGF-*β*1 serum levels are connected with prognostic values for ventricular remodeling and hypertrophy [23, 45] and LV function [24]. Our results showed that the SNT treatment groups had decreased levels of the collagens in sera and in myocardial tissues. There were no significant differences between TGF-*β*1 levels in serum in all experimental groups compared to the sham group. We could show that the mean level of TGF-*β*1 in myocardial tissue was lower in the SNT-HD groups than in the model group, but no significant difference was observed. These data indicated that SNT had a positive influence on the col-I and col-III regulation and therefore had a direct effect on the fibrosis formation and provided structural integrity after AMI. SNT reduced collagen matrix accumulation in the serum and the myocardial tissue, which is associated with a significant improvement in systolic function.

TLRs belong to a group of type I transmembrane receptors with endogenous and exogenous ligand binding ability to stimulate innate and adaptive immune responses by inducing the immune and inflammatory cytokines IL-6, TNF-alpha, and other genes. TLRs are expressed differentially in immune cells and nonimmune cells, such as cardiomyocytes and endothelial cells in the heart that are involved in cardiac stress reactions [46]. TLR-2 signaling is involved in myocardial ischemia/reperfusion injury [21] and in coronary artery endothelial dysfunction with impaired vessel relaxation induced by transient ischemia [47]. TLR-4 plays an important role in mediating immune cells infiltration, cytokine production, and complement activation during ischemia/reperfusion [48]. TLR-4 deficiency improves left ventricular function and attenuates pathophysiological key mechanisms in cardiomyopathy [25]. We could show that the expressions of TLR-2 and TLR-4 were decreased significantly in both SNT treatment groups compared to the sham and the model groups. These findings indicate again that SNT treatment could improve the early left ventricular function after MI.

The emerging understanding of the extracellular matrix and the various active molecules within it, such as the matrix metalloproteinases (MMPs), elicits new appreciation for their role in cardiac remodeling and as possible future therapeutic targets. With further understanding of the complex interaction between MMPs and their temporal activation in the postinfarction heart, pharmacological or transgenic inhibition of MMPs or activation of TIMPs may still prove to be a viable therapeutic option to minimize cardiac remodeling [22]. This should be one of the next steps of our investigation on SNT by omics technologies.

## 5. Conclusions

In conclusion, the present study demonstrates that the Chinese medicine formula SNT has the potential to improve early ventricular remodeling and cardiac function after MI in rats. SNT reduced the left ventricle end-systolic dimension, increased the ejection fraction, and improved the left ventricular function. These effects may be related to the downregulation of Toll-like receptors and collagen deposition. The new insight underlying cardiac remodeling will enable us to develop more successful therapies including the traditional Chinese herbal medicine SNT in modern applications in the future.

## Figures and Tables

**Table 1:** The five experimental groups. Drugs diluted with distilled water were administered orally once a day for 30 days starting two days after induction of AMI.

Group	*N*	Operation	Oral administration
Sham	10	Sham	Drinking water
Model	10	AMI	Drinking water
FS	10	AMI	Fosinopril sodium (0.9 mg/kg)
SNT-LD	10	AMI	Low dose of SNT (4.5 g/kg)
SNT-HD	10	AMI	High dose of SNT (13.5 g/kg)

**Table 2:** Echocardiographic parameters—overview of ventricular remodeling effects.

	Sham	Model	FS	SNT-LD	SNT-HD	Remodeling effects (versus model)
LVDd (mm)	3.29 ± 0.81	6.24 ± 0.72	4.45 ± 1.28**	5.37 ± 1.78	4.87 ± 1.47	↓
LVDs (mm)	0.91 ± 0.20	6.44 ± 1.59**	3.81 ± 1.21^∗∗▲▲^	4.95 ± 1.95**	3.74 ± 1.47^∗∗▲▲^	↓
EF (%)	93.32 ± 2.94	55.48 ± 12.89**	78.03 ± 10.70^∗▲▲^	69.69 ± 13.91^∗▲^	77.83 ± 12.32^∗∗▲▲^	↑
IS (mm^2^)		38.04 ± 8.35	23.91 ± 7.99^▲▲^	31.25 ± 10.68	27.81 ± 4.91^▲^	↓
HW/BW (mg/g)	0.31 ± 0.01	0.37 ± 0.07	0.36 ± 0.08	0.35 ± 0.08	0.36 ± 0.07	↓
LVAC/HCA (%)	10.82 ± 3.01	22.30 ± 3.92**	15.17 ± 6.54^▲▲^	18.48 ± 3.96*	15.25 ± 2.78^▲^	↓

*P < 0.05, **P < 0.01 versus sham group; ^▲^ *P* < 0.05, ^▲▲^ *P* < 0.01 versus model group.

**Table 3:** Overview of TLR expression levels in myocardial tissue.

	Sham	Model	FS	SNT-LD	SNT-HD	Expression levels (versus model)
In myocardial tissue						
TLR-2 (IOD)	896.40 ± 89.18	4254.60 ± 413.66**	3381.00 ± 432.17^∗∗▲▲^	3646.60 ± 362.37**	3316.60 ± 439.06^∗∗▲▲^	↓
TLR-4 (IOD)	973.00 ± 95.13	5184.40 ± 566.30**	3470.60 ± 403.33^∗∗▲▲^	4378.60 ± 748.49**	3732.20 ± 528.23^∗∗▲▲^	↓

IOD: integral optical density. **P < 0.01 versus sham group; ^▲▲^ *P* < 0.01 versus model group.

**Table 4:** Overview of collagen expression levels in serum and in myocardial tissue.

	Sham	Model	FS	SNT-LD	SNT-HD	Expression levels (versus model)
In serum						
Collagen I (*μ*g/L)	2.74 ± 0.40	4.11 ± 0.74**	2.67 ± 0.42^▲▲^	2.43 ± 0.38^▲▲^	2.17 ± 0.21^▲▲^	↓
Collagen III (*μ*g/L)	1.61 ± 0.27	2.68 ± 0.43**	2.00 ± 0.45^▲▲^	2.02 ± 0.47^▲▲^	1.96 ± 0.47^▲▲^	↓
TGF-*β*1 (ng/L)	24.95 ± 4.31	23.91 ± 5.76	22.84 ± 5.07	23.75 ± 8.78	25.61 ± 7.98	
In myocardial tissue						
Collagen I (*μ*g/g)	0.94 ± 0.16	1.20 ± 0.15	0.86 ± 0.15^▲^	1.03 ± 0.10	0.89 ± 0.14^▲^	↓
Collagen III (*μ*g/g)	0.28 ± 0.10	0.45 ± 0.04*	0.33 ± 0.04	0.36 ± 0.08	0.30 ± 0.04^▲^	↓
TGF-*β*1 (ng/g)	5.99 ± 2.17	6.48 ± 1.79	5.91 ± 2.80	5.32 ± 0.49	4.08 ± 1.05	↓

*P < 0.05, **P < 0.01 versus sham group; ^▲^ *P* < 0.05, ^▲▲^ *P* < 0.01 versus model group.
